# Hematologic malignancies following immune checkpoint inhibition for solid tumors

**DOI:** 10.1007/s00262-022-03230-4

**Published:** 2022-06-13

**Authors:** Mick J. M. van Eijs, Lotte E. van der Wagen, Rogier Mous, Roos J. Leguit, Lisette van de Corput, Anne S. R. van Lindert, Britt B. M. Suelmann, Anna M. Kamphuis, Stefan Nierkens, Karijn P. M. Suijkerbuijk

**Affiliations:** 1 Department of medical oncology, University Medical Center Utrecht, P.O. Box 85500, 3508 GA Utrecht, The Netherlands; 2 Center for Translational Immunology, University Medical Center Utrecht, Utrecht, The Netherlands; 3 Department of hematology, University Medical Center Utrecht, Utrecht, The Netherlands; 4 Department of pathology, University Medical Center Utrecht, Utrecht, The Netherlands; 5 Central Diagnostic Laboratory, University Medical Center Utrecht, Utrecht, The Netherlands; 6 Department of pulmonology, University Medical Center Utrecht, Utrecht, The Netherlands; 7 Princess Máxima Center for Pediatric Oncology, Utrecht, The Netherlands

**Keywords:** Case series, Immune checkpoint inhibitor, Leukemia, Myeloid neoplasms, Second primary cancer

## Abstract

Immune checkpoint inhibition (ICI) can induce durable responses in patients with advanced malignancies. Three cases of hematological neoplasia following ICI for solid tumors have been reported to date. We present five patients treated at our tertiary referral center between 2017 and 2021 who developed chronic myeloid leukemia (two patients), acute myeloid leukemia, myelodysplastic syndrome and chronic eosinophilic leukemia during or after anti-PD-1-based treatment. Molecular analyses were performed on pre-ICI samples to identify baseline variants in myeloid genes. We hypothesize that PD-1 blockade might accelerate progression to overt myeloid malignancies and discuss potential underlying mechanisms.

## Introduction

Immune checkpoint inhibition (ICI) targeting cytotoxic T-lymphocyte antigen 4 (CTLA-4) or programmed cell death [ligand] 1 (PD-[L]1) can induce durable responses in some patients with advanced malignancies. A well-established downside of ICI is its diverse spectrum of immune-related adverse events (irAEs). If ICI therapy reduces or increases second primary malignancy incidence, has been a matter of debate [1, 2]. Here, we present five patients treated at our academic center between 2017 and 2021 who developed clinically manifest myeloid neoplasia during or after ICI treatment for solid tumors. Two patients developed chronic myeloid leukemia (CML), one acute myeloid leukemia (AML), one myelodysplastic syndrome (MDS) and one chronic eosinophilic leukemia (CEL). For three patients we performed molecular analyses on baseline material, to identify baseline variants in myeloid genes. Lastly, we discuss these findings in the light of possible links between ICI and the subsequent manifestation of myeloid neoplasia, along with alternative explanations.

## Case series

**Fig. 1:**
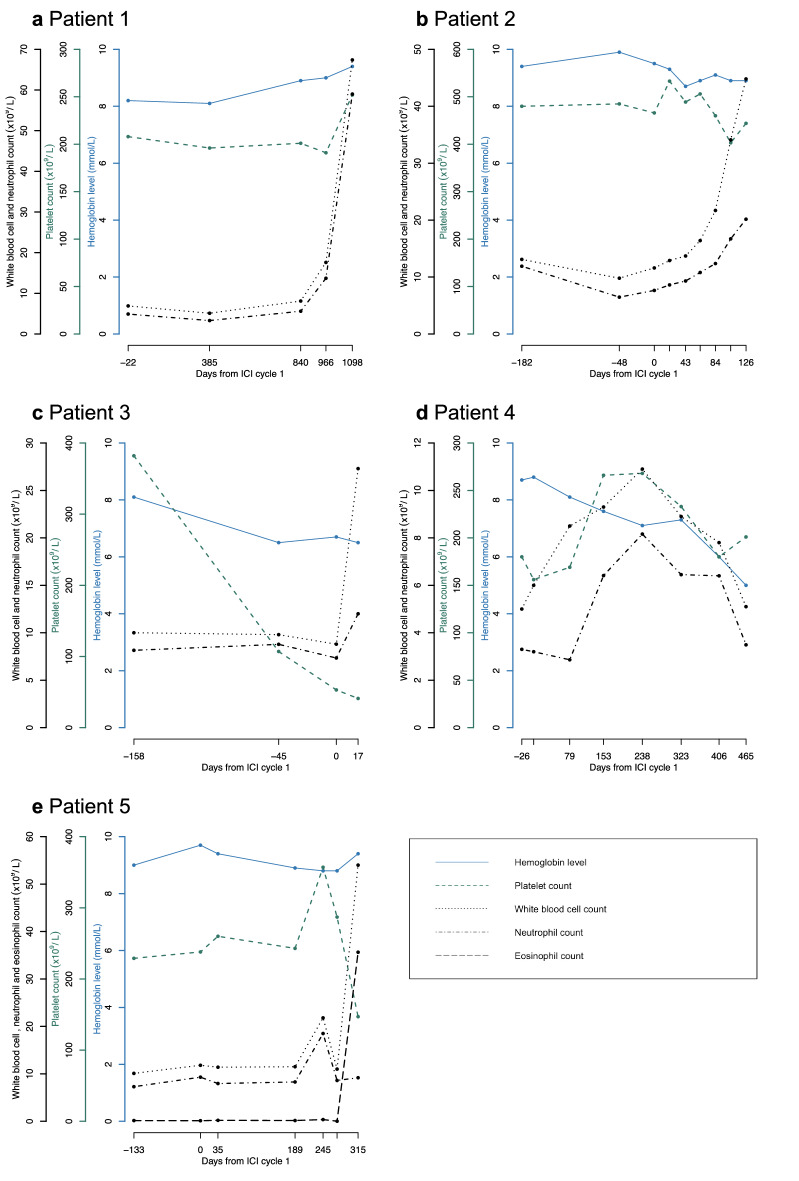
**a**–**e** Course of blood counts for patients 1–5, relative to the first cycle of ICI (‘day 0’). In patient 3, day ‘0’ marks the first cycle of nivolumab; not the first cycle of priorly administered durvalumab

Clinical characteristics and complete blood counts (CBC) are displayed in Table 1 and Fig. 1, respectively.

**Table 1:** Clinical characteristics of patients

	Patient 1	Patient 2	Patient 3	Patient 4	Patient 5
Sex	Male	Male	Female	Male	Male
Age decade, yr	60s	50s	70s	60s	80s
Alive	yes	no	no	yes	no
*Primary malignancy*					
Type	Melanoma	Melanoma	NSCLC	Melanoma	ccRCC
Stage	IV	IV	IV	IV	IV
*CBC (reference values) before ICI*					
Hemoglobin (7.4/8.6–10.7), mmol/L	8.2	9.5	8.1^*^	8.8	9.4
WBC count (4.0–10.0), × 10^9^/L	6.9	11.6	10.2^*^	6.0	11.4
Platelet count (150–450), × 10^9^/L	208	466	342^*^	156	260
*Cancer treatment*					
ICI treatment					
Type	Pembrolizumab	Pembrolizumab	Nivolumab	Nivolumab (maintenance)	Nivolumab (maintenance)
Total *N* cycles	15	6	2	10 (after 4 × combined ipi/nivo)	3 (after 4 × combined ipi/nivo)
Setting	Palliative	Palliative	Palliative	Palliative	Palliative
On/off-treatment upon 2^nd^ neoplasm	Off-treatment	On-treatment	On-treatment	On-treatment	On-treatment
Reason ICI cessation	Durable response	N/A	N/A	N/A	N/A
Best overall response	Partial response	Complete response	Not evaluable	Stable disease	Partial response
Previous treatment					
ICI (cycles)	Ipilimumab (1)	–	Durvalumab (12)	–	–
Chemotherapy (cycles)	Dacarbazine (4)	–	Cisplatin/etoposide (2)	–	–
			Carboplatin/pemetrexed (3)		
Irradiation	–	Yes (brain)	Yes	Yes (brain)	Yes
*Subsequent neoplasia*					
Diagnosis	CML	CML	AML	MDS-EB1	CEL-NOS
Driver/gatekeeper mutations (VAF)	*BCR-ABL1*	*BCR-ABL1*	*NRAS *(9%)*, RUNX1 *(6.4%)	Not tested	*SRSF2 *(40%)*, NPM1 *(35%)
Time since start latest ICI, weeks	148	18	58	58	42
Treatment	Dasatinib	Nilotinib	Best supportive care	Best supportive care, darbepoetin	Hydroxyurea, dasatinib, dexamethasone
Response	Optimal response	Optimal response	N/A	N/A	Not responding
*irAEs (CTCAE v5.0 grade)*					
Concomitant with 2nd neoplasm	–	Arthritis (II)	–	–	PMR flare (II)
Previous	–	–	–	Dermatitis (II), hypophysitis (III), myalgia (I), arthralgia (I)	Arthritis (II), colitis (I), dermatitis (II), PMR (II)

*AML* denotes acute myeloid leukemia, *CBC* complete blood count, *ccRCC* clear cell renal cell carcinoma, *CEL-NOS* chronic eosinophilic leukemia, not otherwise specified, *CML* chronic myeloid leukemia, *CTCAE* common terminology criteria for adverse events, *EPO* erythropoietin, *ICI* immune checkpoint inhibition, *irAE* immune related adverse event, *HES* hypereosinophilic syndrome, *MDS-EB* myelodysplastic syndrome with excess blasts, *N/A* not applicable, *NSCLC* non-small cell lung cancer, *PMR* polymyalgia rheumatica, *VAF* variant allele frequency, *WBC* white blood cell

^*^CBC from before initiation of last chemotherapeutic regimen before palliative nivolumab, because chemotherapy led to myelodepression

Patient 1 is a man in mid-60s with a history of anti-CCP-negative rheumatoid arthritis and ulcerative colitis in remission without treatment. He developed stage IV melanoma which progressed after dacarbazine long ago and was treated with one cycle of ipilimumab, followed by pembrolizumab for 11 months [3], resulting in good partial response. Two years after pembrolizumab discontinuation, leukocytosis (67.4 × 10^9^/L) was found (hemoglobin level [Hb] 9.4 mmol/L, platelet count [PLT] 255 × 10^9^/L). Bone marrow biopsy showed marked granulocytic proliferation with < 1% blasts. Fluorescence in situ hybridization (FISH) and reverse transcription polymerase chain reaction (RT-PCR) detected the *BCR-ABL1* fusion gene, without additional cytogenetic aberrations. CML (low Sokal score) was diagnosed and, eventually, dasatinib 100 mg q.d. resulted in optimal response. During three-year follow-up, optimal response to dasatinib persisted, without melanoma progression. FISH performed on lymphocytes (since granulocytes were scarce) from a paraffin-embedded lymph node biopsy obtained before pembrolizumab initiation did not reveal *BCR-ABL1*.

Patient 2, a man in mid-50s, was diagnosed with stage IV *BRAF* mutant melanoma with brain and liver metastases. After resection and radiotherapy of the brain metastasis, pembrolizumab was started. Upon initiation of pembrolizumab, leukocytes and platelets were 11.6 × 10^9^/L and 446 × 10^9^/L, respectively. After four months, patient developed leukocytosis (44.8 × 10^9^/L) with normal differentiation (Hb 7.9 mmol/L, PLT 444 × 10^9^/L). With scans showing complete response of the melanoma metastases, pembrolizumab was stopped after six cycles. FISH and RT-PCR for *BCR-ABL1* were positive, without additional cytogenetic abnormalities. The bone marrow was hypercellular with marked granulocytic proliferation, decreased erythropoiesis, small megakaryocytes, and < 5% blasts, supporting the diagnosis of CML (low Sokal score). Although the CML remained in durable remission under nilotinib treatment, patient died one year later from progressive melanoma with leptomeningeal metastases. No *BCR-ABL1* fusion could be detected through FISH in granulocytes from a paraffin-embedded brain biopsy obtained pre-ICI.

Patient 3 is a female in mid-70s known with non-small cell lung cancer (NSCLC) for 12 years. After initial radical resection, followed by stereotactic radiotherapy (60 Gy) on a solitary lesion, concurrent chemoradiotherapy (cisplatin/etoposide with 30 × 2 Gy) and durvalumab for a stage IIIB relapse were initiated. Six months later, she underwent a lobectomy with adjuvant chemotherapy (carboplatin/pemetrexed) for clonally unrelated second NSCLC. After three cycles, chemotherapy was stopped for intolerance. Anemia (Hb 5.7–7.3 mmol/L) and thrombocytopenia (PLT ± 100 × 10^9^/L) incompletely recovered after chemotherapy cessation. Because of rapid disease progression, nivolumab monotherapy was initiated, with known anemia, thrombocytopenia and leukocytosis upon cycle 1. Leukocyte differentiation revealed mainly segmented neutrophils, 11% monocytes, 1% myelocytes and promyelocytes, but no blasts. Two weeks later, before the second nivolumab cycle, leukocytosis (27.3 × 10^9^/L) was found with 39% monocytes, and 39% segmented neutrophils and 6% blasts containing Auer rods. Blood immunophenotyping revealed 10% myeloid blasts. AML or MDS with excess blasts (MDS-EB)-2 was suspected, for which the patient wished neither diagnostics nor treatment. After several weeks she succumbed. Molecular analysis by next-generation sequencing (NGS) of a preserved cell pellet from the time of AML onset revealed pathogenic variants in *NRAS* (VAF 9%) and *RUNX1* (VAF 6.4%), and a variant of unknown significance (VUS) in *CEBPA* (VAF 6%). NGS analysis of PBMCs collected, while 1.5 months on durvalumab treatment demonstrated 100% wild-type allele frequencies of all three genes.

Patient 4 is a man in mid-60s with metastatic melanoma for which combined ipilimumab and nivolumab were started. At immunotherapy initiation, CBC was unremarkable (leukocytes 6.0 × 10^9^/L, Hb 8.8 mmol/L, PLT 156 × 10^9^/L). Stable disease was achieved after 4 cycles, while over the course of one year anemia slowly developed (Hb 6.1 mmol/L upon nivolumab cycle 11). Bone marrow investigation demonstrated trilinear dysplasia and 9% CD34^+^ blasts. MDS-EB1 was concluded with a minimal R-ISS score of 4 (intermediate risk). Given ineligibility for allogeneic stem cell transplantation, cytogenetic evaluation was waived. Instead, best supportive care including subcutaneous darbepoetin 150 µg/week was initiated and seven weeks later increased to 300 µg/week for persisting anemia (Hb ≤ 5.5 mmol/L).

Patient 5, a man in his 80s, had a history of polymyalgia rheumatica, for which he used 10 mg prednisone daily, and metastatic clear-cell renal cell carcinoma (without pulmonary metastases) for which combined ipilimumab and nivolumab were started. Ten months afterward, he presented at the emergency department with progressive dyspnea. Laboratory analysis revealed severe hypereosinophilia (35.6 × 10^9^/L), with Hb 9.4 mmol/L, PLT 147 × 10^9^/L, 9.18 × 10^9^/L segmented neutrophils, 0.54 × 10^9^/L basophils, 1.62 × 10^9^/L monocytes and 6.48 × 10^9^/L lymphocytes. Since eosinophilia is frequently seen in the context of irAEs, prednisone 2 mg/kg was pragmatically started. Nevertheless, eosinophils rose to 104 × 10^9^/L over several days and first decreased after the start of hydroxycarbamide. Blood and bone marrow immunophenotyping revealed 0.65% and 0.40% monoclonal IgM lambda-positive B cells, respectively, suspect for hairy cell leukemia variant. However, related lymphocytic hypereosinophilic syndrome (HES) was deemed unlikely given absence of a T cell clone and low serum interleukin-5 and G-CSF. The bone marrow was hypercellular (85% cellularity) due to marked eosinophilic proliferation without blast increase. T lymphocytes were normal. *SRSF2* (VAF 40%) and *NPM1* mutations (VAF 35%) were found and imatinib, later replaced by dasatinib, was added for suspected CEL not otherwise specified. Despite further decrease following tyrosine kinase inhibitors (TKIs), eosinophils did not reach the normal range. After initiation of anticoagulants for newly developed pulmonary embolisms, hydroxycarbamide was discontinued. With alleged unavoidable short-term progression to AML [4], patient was referred to a hospice. Remarkably, two weeks after hospice admission he recovered clinically. Seven weeks later, while on steroids, leukocytosis had improved (14 × 10^9^/L). Shortly after, patient died of complicated COVID-19 infection.

## Discussion

Between 2017 and 2021, we encountered five cases of myeloid neoplasia developing during or shortly after ICI treatment (PD-[L]1 inhibitors with or without CTLA-4 inhibitors). A pharmacovigilance study previously detected no statistically significant disproportionality signal for AML or CML and ICI [5].

Alternative mechanisms should be considered. Etoposide exposure 19 months before AML in the third patient suggests a chemotherapy-induced etiology [6]. Nevertheless, in this case the acute leukemic blast crisis, remarkably, developed immediately after the first cycle of nivolumab. Regarding patient 1, CML has not been associated with prior chemotherapy exposure and secondary cancer incidence was shown not to be increased after dacarbazine in the context of the ABVD regimen without irradiation for Hodgkin’s lymphoma [7]. Although HES has been described as an immune-related phenomenon, peak eosinophil counts in previous cases were significantly lower than in patient 5 [8]. Moreover, previous HES patients demanding pharmacological management generally showed rapid responses to steroids [8], whereas patient 5 required hydroxycarbamide and TKIs beyond steroids with the first decrease in eosinophils after 8 days. Together with proven driver mutations [4], we therefore considered CEL-NOS more likely than a severe irAE.

We found no pre-ICI molecular aberrations in patients 1–3. This could indicate that at baseline pathogenic mutations were absent in all three patients and malignant transformation had yet to occur upon durvalumab initiation in patient 3. However, for the lack of preferential baseline biospecimens, low cell yield and the absence of bone marrow samples, these analyses cannot rule out myeloid somatic variants or the presence of subclinical malignant clones at baseline. Importantly, results do not preclude the possibility that ICI contributes to shorter time-to-manifestation of myeloid neoplasia.

Both preclinical [9] and clinical [10, 11] evidence support that PD-1 blockade can lead to secondary lymphoproliferative disease. Indeed, one hypothesis suggests that mutant T cell clones are provided with an additional proliferation advantage following lymphoid PD-1 blockade [10]. However, regarding myeloid lineage, myeloid-specific PD-1 ablation in a murine melanoma model *enhanced* effector T response and led to a larger decrease in tumor growth than T-cell-specific PD-1 ablation [12]. Yet, ICI mechanisms of action have proven complex and many theories explaining non-response or hyperprogression have been developed, including ‘tumor-intrinsic PD-1 signaling.’ Kim et al. [13] reported a case of acute myelomonocytic leukemia (AMML) after three cycles of pembrolizumab for NSCLC. The authors hypothesized hyperprogression of subclinical AMML as a potential explanation. In such theory, a myeloid clone with acquired driver mutation(s) could obtain an extra proliferation advantage from functional myeloid PD-1 knockout after ICI. Analogically, in CML the *BCR-ABL1* fusion is the only mandatory molecular criterion for CML. Additional events, however, are probably necessary for progression to overt CML, since some healthy individuals carry *BCR-ABL1* [14].

*PDCD1* transcription has been demonstrated in over 30 cancers, including hematological malignancies [15]. Surface PD-1 expression could be confirmed in subsets of cells in 40 different cancer cell lines by flow cytometry [15]. Specifically, abberant PD-1 expression has been demonstrated in 8–26% of CD34^+^ blasts in MDS, CMML and AML [16]. Although the latter study found no difference in AML/MDS overall survival depending on PD-1 expression in blasts [16], another study in AML reported that higher PD-1 expression on leukemic blasts was associated with longer disease-free survival [17]. In MDS, an approximately fourfold larger fraction of CD34^+^ blasts was PD-1^+^ compared to healthy control bone marrow [18]. This upregulation of the PD-1/PD-L1 axis was shown to contribute to premature death of hematopoietic progenitors in MDS, while hematopoiesis could be recovered by PD-1/PD-L1 blockade in aged S100A9 transgenic mice (which phenotypically resemble MDS) and human MDS bone marrow mononuclear cells [18]. Although such restoration of HSC survival may appear favorable, as it presents possibilities for MDS treatment, it could also contribute to clonal selection of malignantly transformed progenitors.

In T lymphocytes, PD-1/PD-L1/2 ligation results in SHP-2 binding, followed by CD28 and T cell receptor dephosphorylation [19]. In contrast, non-canonical PD-1 signaling has been demonstrated in cancer cells, resulting in both protumor and tumor-suppressing effects [19]. Protumor effects of intrinsic PD-1 have been shown through multiple mechanisms including enhanced mTOR signaling and NFκB activation in some tumors and may even act indepently of PD-1/PD-L1 ligation [20–23]. On the contrary, in vitro and in vivo* PDCD1* knockdown models, as well as immunodeficient mice inoculated with lung cancer cell lines and treated with anti-PD-1, showed increased colony expansion and tumor growth [15, 24], mediated through upregulated AKT and ERK signaling [15]. Along these lines, p53 has been shown to transcriptionally regulate PD-1 expression in cancer cells and induced cancer cell intrinsic PD-1 expression could inhibit tumor growth in mice [25]. These experimental data support the hypothesis that anti-PD-1 may functionally knock-out tumor-intrisic PD-1 and lead to accelarated clonal expansion of myeloid malignant cells.

Besides tumor intrinsic PD-1 signaling, the inflammatory milieu may also mediate clonal expansion through ICI-induced cytokines or chemokines that sort effects in particular myeloid subsets. In acute inflammation, IFN-*γ* and GM-CSF increase myeloid differentiation bias and chronic inflammation may pose selective pressure to premalignant clones [26]. Elevated IL-16 and CCL2 after ICI treatment have been associated with increased eosinophil accumulation [27]. However, the complexity of cytokine responses following ICI, in rare cases escalating to cytokine release syndrome [28], prevents definite statements on which effects cytokines have on myeloid clonal selection.

In conclusion, PD-1 blockade might accelerate progression to overt myeloid malignancies, for example in one of the above-hypothesized ways. Although we could not elucidate the exact mechanism, the increasing number of patients treated with ICIs and incomplete understanding of ICI mechanisms warrants attention for the above cases. Prospective studies into this particular matter will not be feasible given relatively low incidence and required follow-up. Instead, pharmacovigilance studies, preclinical models and studies examining immune response after ICI can help reveal if a particular mechanism underlies our clinical observations.

## Abbreviations

Hb: Hemoglobin level
ICI: Immune checkpoint inhibition
irAE: Immune-related adverse event
PLT: Platelet count
TKI: Tyrosine kinase inhibitor
VAF: Variant allele frequency
